# Buildings Contemplated

**Published:** 1914-02-28

**Authors:** 


### Buildings Contemplated and in Progress.

Bradford.—The Corporation has passed a resolution agreeing to purchase the site of the present Royal Infirmary and buildings for the sum of £100,000.

Bromsgrove.—At a recent meeting of the Worcestershire County Council it was announced that the Board of Control under the Mental Deficiency Act desired to negotiate for the purchase of the mental hospital at Barnsley Hall, near Bromsgrove. The Council has resolved to appoint a committee to conduct negotiations.

Burton-on-Trent.—The Board of Guardians are inviting competitive designs for children's cottage homes, which it is proposed to erect at Burton-on-Trent. Premiums of £25 and £10 respectively as first and second prizes. Full particulars may be obtained from the Clerk to the Guardians, to whom designs are to be delivered not later than March 25.

Frome.—The Urban District Council has resolved to extend and improve the Victoria Hospital at an estimated cost of £1,250.

Glen Lomond.—The Convener's Committee of the Fife County Council has approved the site of the proposed sanatorium at Glen Lomond.

Ipswich.—The Guardians are taking up a loan of £8,600 for the purpose of extending the workhouse.

Cheshire.—The County Council has resolved to provide a central dispensary at Chester, with branch dispensaries at Tarporley, Ellesmere Port, West Kirby, and Birkenhead; a central dispensary at Crewe, with branch dispensaries at Winsford, Middlewich, and Congleton; and central dispensaries at Hyde and Northwich, with sub-dispensaries at Runcorn and Altrincham respectively.

Hitchin.—The Rural District Council has resolved to apply to the Local Government Board for sanction to borrow £8,950 for a new isolation hospital.

Kingseat.—The Aberdeen City Lunacy Board has resolved to extend the mental hospital at Kingseat.

Middlesbrough.—The Borough Engineer, Mr. S. E. Burgess, has prepared plans for the extension of the Middlesbrough Mental Hospital to increase the accommodation by sixty beds. The estimated cost of the building and furnishing is £7,952.

Norton.—The Stockton Guardians have resolved to apply to the Local Government Board for sanction to borrow £1,750 for the purchase of a site for a new infirmary.

Paignton.—The Urban District Council has decided to apply to the Local Government Board for sanction to borrow £2,537 for additions to the hospital.

